# IFN-β induces greater antiproliferative and proapoptotic effects and increased p53 signaling compared with IFN-α in PBMCs of Adult T-cell Leukemia/Lymphoma patients

**DOI:** 10.1038/bcj.2016.126

**Published:** 2017-01-27

**Authors:** T Dierckx, R Khouri, S M Menezes, D Decanine, L Farre, A Bittencourt, A M Vandamme, J Van Weyenbergh

**Affiliations:** 1 Department of Microbiology and Immunology, Rega Institute for Medical Research, Laboratory for Clinical and Epidemiological Virology, KU Leuven - University of Leuven, Leuven, Belgium; 2 Instituto Gonçalo Moniz-FIOCRUZ, Salvador-Bahia, Brazil; 3 Hospital Universitário Professor Edgar Santos-UFBA, Salvador-BA, Brazil; 4 Center for Global Health and Tropical Medicine, Unidade de Microbiologia, Instituto de Higiene e Medicina Tropical, Universidade Nova de Lisboa, Lisbon, Portugal

Adult T-cell Leukemia/Lymphoma (ATLL) is an aggressive T-cell malignancy with a poorly understood pathology that manifests from human T-lymphotropic virus type I (HTLV-1) infected T-cells, typically after long latency periods (>30 years). Current treatment regimens for ATLL include zidovudine (AZT) and interferon alpha (IFN-α) combination therapy.^1^ The established usage of IFN-α in the treatment of ATLL is largely empirical in origin. The effects of the other widely used IFN subtype, IFN-β, have not been thoroughly examined in this setting, even though IFN-β's more potent induction of antiproliferative and apoptotic pathways has been described in solid cancers.^2^ Few studies have compared the effects of IFN-α versus those of IFN-β in ATLL and these were generally performed in cell lines, rather than primary patient cells.^3, 4^ *In vitro* experiments with IFN-α using cell lines report minimal effects of IFN-α on the viability of HTLV-1-infected T-cells and viral replication, in contrast to the high *in vivo* response rates obtained by this therapy.^5, 6^ Reports regarding *in vitro* and *in vivo* activity of IFN-β in this context also seem contradictory: *in vitro* experiments showed no antiproliferative action of IFN-β,^7^ while a single early *in vivo* trial using IFN-β reported promising results, with 50% of six treated patients achieving partial response to treatment using IFN-β monotherapy.^8^ The reported success rate of this IFN-β monotherapy was comparable to early AZT/IFN-α combination therapy trials where 67% of 24 treated patients achieved partial response.^9^

We performed the first direct comparison between the response to IFN-α and IFN-β in *ex vivo* ATLL patient PBMCs. We analyzed samples obtained between 2001 and 2007 from 9 men and 13 women aged 21–78 years (median 47.5), diagnosed as HIV negative and definite ATLL with serology, inverted PCR and/or flow cytometry, at the ‘Hospital Universitário Professor Edgar Santos' (HUPES) in Salvador, Bahia, Brazil. Seven of these patients were classified as acute, ten as smoldering, three as chronic and two as lymphoma according to Shimoyama criteria.^10^ This study was approved by the Ethics Review Board of HUPES (number 32050106). Data handling and processing was additionally approved by the Medical Ethics Commission of the UZ Leuven hospital, Belgium (number s57931).

Proliferation, antiviral activity and apoptosis were all measured in three distinct treatment conditions: cultures were either left untreated or stimulated with either IFN-α (1000 U/ml) or IFN-β (1000 U/ml) at the start of the experiment. Bioactivity of IFN-α and IFN-β was determined using a VSV/Wish bioassay in order to preclude any bias owing to different antiviral effects of the interferon subtypes. Neither IL-2 nor PHA were added to the *ex vivo* cultures in order to approximate *in vivo* conditions as closely as possible. Proliferation was measured by [^3^H] thymidine incorporation assay in the cultures of *ex vivo* PBMCs of 19 patients. Active caspase-3 was measured by flow cytometry (FACSort, BD Biosciences, Franklin Lakes, NJ, USA) using a CBA apoptosis kit (BD Biosciences). HTLV p19 protein levels in PBMC 48-h culture supernatants were measured using the HTLV-I/II p19 antigen ELISA (ZeptoMetrix, Buffalo, NY, USA), according to the manufacturer's instructions. Detailed methods as well as all experimental results are provided as supplementary materials.

Unless otherwise noted, Bonferroni-corrected, nonparametric Friedman rank sum tests were used to test for statistically significant differences between the three experimental conditions. The results of these tests are summarized in Figure 1. IFN-α caused a small but significant 24±36% (mean±s.d.) decrease in proliferation in the 19 examined samples, whereas IFN-β treatment decreased proliferation significantly by 47±58% (mean±s.d.). Direct comparison of IFN-α vs IFN-β treatment conditions shows that IFN-β exerted superior antiproliferative activity. Caspase 3 activation, measured in six samples, showed an increase in apoptosis for both IFN subtypes, but IFN-β showed a significantly higher increase in apoptosis than IFN-α (12.8±7.2 and 4.9±7.1 pg/ml, mean±s.d., respectively). Fourteen out of 16 tested samples had detectable virus production in the supernatants of 48-h cultures. Viral p19 levels varied strongly between patient samples, ranging from 4.8 to 10792.7 pg/ml (mean±s.d., 2131.3±3796.9) in the control condition. Both IFN-α and IFN-β treatments resulted in comparable reductions in viral p19 levels when contrasted with the untreated control condition (a mean±s.d. decrease of 49±32% versus 69±70%), suggesting that the observed differential effects of the two IFN types on proliferation and apoptosis do not stem from a differential impact on viral replication.

Sample quantities did not allow for the purification of leukemic cells, so their relative contribution to the composition of the examined PBMCs could not be determined. Additionally, at the time of patient recruitment and sample processing for *ex vivo* experiments, TSLC1/CADM1 had not yet been described as a reliable flow cytometry marker for HTLV-1 infected and ATLL leukemic cells.^11, 12^ As such, we could not determine whether the observed differential effects of IFN-α and IFN-β take place in leukemic, HTLV-1 infected non-leukemic or HTLV-1-negative cells. However, in a re-analysis of the data comparing the effects of IFN-α and IFN-β on proliferation in ATLL subtypes with high percentages of circulating ATLL cells (that is, acute and chronic subtypes, *n*=8), the differences remained significant (*P*=0.004). In contrast, patients with low percentages of circulating ATLL cells (that is, lymphoma and smoldering subtypes, *n*=11) did not show these significant differences (*P*=0.36), suggesting that the differential effects occur in the leukemic cells. Viral p19 protein measurements show no significant differences between IFN-α and IFN-β treatments in these subgroups (*P*=0.32 and *P*=0.71 for high and low ATLL cell percentage subgroups, respectively).

These findings were complemented with microarray analysis. Both the preprocessed and the raw data from the microarray experiments are available at the National Center for Biotechnology Information Gene Expression Omnibus under accession number GSE85487. Paired differential expression analysis comparing control, IFN-α- and IFN-β-treated samples of six patients shows that the IFN-β response can be regarded as both broader and stronger than the IFN-α response: all but two of the significantly IFN-α regulated genes were regulated more strongly by IFN-β (one-sided, paired *t*-test, *P*<0.005).

Although the mechanism behind the AZT/IFN-α treatment in ATLL has not been fully elucidated,^6, 13^ recent research pointed to the protein kinase R (PKR or EIF2AK2) gene as a critical gene in the antiviral response and the activation of the p53 pathway and the induction of apoptosis as key components in its mechanism.^14^ We report a superior effect of IFN-β on all of these processes. First, our microarray results show that IFN-β affects PKR gene transcription more strongly than IFN-α (21 vs 15% increase, *P*=0.04). Second, Gene Set Analysis (GSA) shows that the enrichment of the canonical apoptosis pathway that is prominent in the IFN-β condition is absent in the IFN-α condition (Figure 2). Third, while p53 gene expression (TP53) is not significantly affected by either IFN-α or IFN-β treatment, in agreement with the post-transcriptional stabilization of p53 by AZT,^15^ GSA shows that the p53 pathway is more strongly activated by IFN-β than by IFN-α (Figure 2). Finally, in agreement with the results of Kinpara *et al.*,^14^ GSA of the IFN-α treatment microarray results revealed a modest downregulation of the NF-κB pathway, which has been identified as integral to the ATLL transcriptome in a recent integrated omics analysis in a large number of patients.^16^ In contrast to the IFN-α effects, IFN-β treatment upregulated NF-κB pathway activation. Combined, these results suggest that IFN-β has a greater impact on the crucial elements of the AZT/IFN-α treatment mechanism than IFN-α, already making a strong case for possible clinical trials using IFN-β or AZT/IFN-β. But perhaps the clearest argument in favor of clinical trials using IFN-β in ATLL is that most of the genes in the *in vivo* AZT/IFN-α response gene set reported by Alizadeh *et al.*^13^ respond more strongly to IFN-β mono-stimulus than to IFN-α mono-stimulus in these *ex vivo* PBMCs (Supplementary Table 2).

Other preclinical cancer models have previously shown superior antiproliferative and proapoptotic effects of IFN-β when compared with IFN-α^2, 17^ but, to our knowledge, this is the first time that these differential effects have been reported in *ex vivo* PBMCs of leukemia/lymphoma patients. Due to three strong similarities with observations made by Sancéau *et al.*^17^ in a preclinical Ewing Sarcoma model, we hypothesize that other leukemias with a functional or wild-type p53 could also be more sensitive to the proapoptotic effects of IFN-β as compared with IFN-α. First, the four cell lines examined in their study proved to be more susceptible to the antiproliferative effects of IFN-β than to those of IFN-α, similar to the PBMCs of ATLL patients used in the present report. Second, IFN-β, but not IFN-α, induced apoptosis in the two cell lines with wild-type p53 but not in those with mutant p53,^17^ which is in line with p53's mutant status as a predictor of poor response to AZT/IFN-α treatment^15^ and the p53-dependence AZT/IFN-α-induced apoptosis.^14^ Third, Sancéau *et al.*^17^ showed that IFN-β-induced apoptosis was mediated by IRF1, which is also associated with AZT/IFN-α treatment response in ATLL.^13^

In conclusion, we performed the first comprehensive analysis comparing the effects of IFN-α and IFN-β on *ex vivo* PBMCs of ATLL patients. Our observations suggest that IFN-β is a worthwhile candidate for clinical trials in ATLL and is also worth investigating in other leukemic contexts where IFN-α has shown modest success.

## Figures and Tables

**Figure 1:**
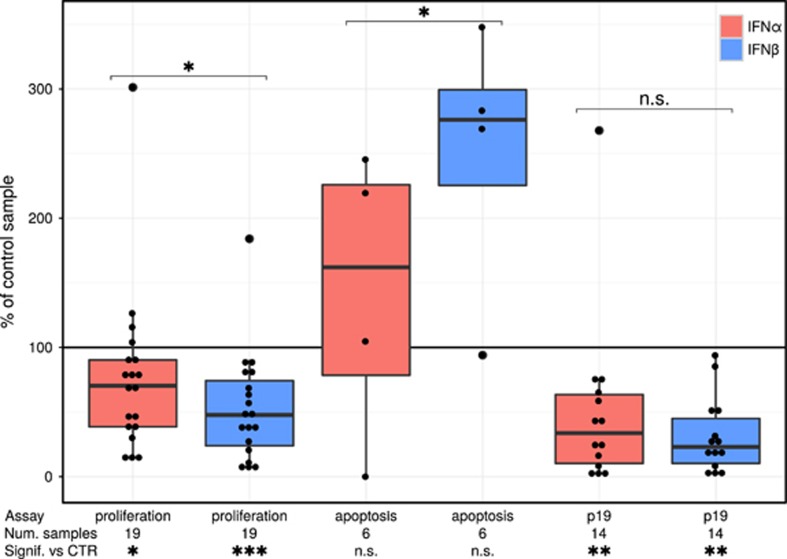
Boxplots of the effects of IFN-α and IFN-β on measured proliferation, apoptosis and viral protein p19 production in *ex vivo* PBMCs of ATL patients. Proliferation was quantified by [^3^H] incorporation, apoptosis through flow cytometry of active caspase-3 and viral protein was quantified using ELISA. Each sample was treated in parallel in three different conditions: either left untreated, stimulated with 1000 IU of IFN-α or IFN-β (red and blue, respectively). The data are depicted here as the percentage of the value measured in the corresponding untreated control condition of the sample. Statistical significance of the Friedman rank sum test (*P<0.05, **P<0.01, ***P<0.001) and number of samples used in the comparison of each condition versus its control (CTR) is indicated underneath the graph. Statistical significance of the difference between IFN-α and IFN-β is depicted above the boxplots. All the datapoints are shown in the graph, excepting two samples that had no measurable apoptosis in the untreated control condition. These could not be included in this graph, but have been used in the statistical comparison of significance versus control.

**Figure 2:**
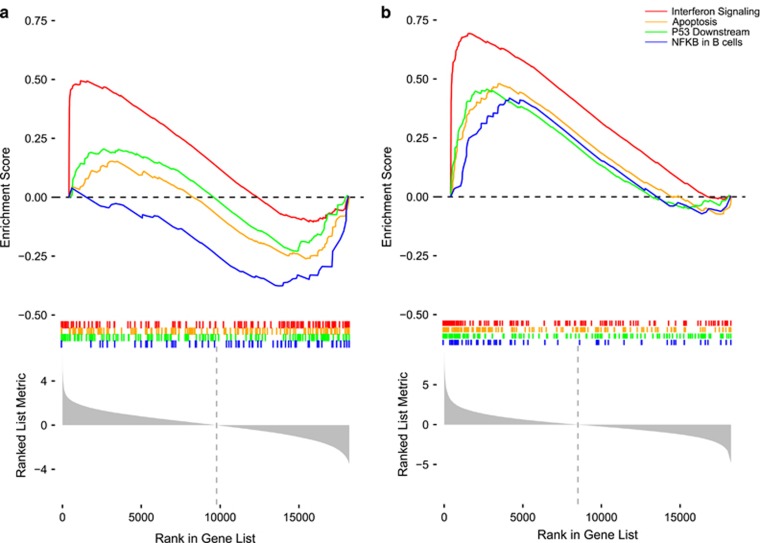
Selected gene set enrichment analysis results. The impact of IFN-α treatment (**a**) and IFN-β treatment (**b**) on four selected gene sets relevant to ATL associated with interferon signaling (red), apoptosis (yellow), p53 signaling (green) and NF-κB signaling (blue). The top graph depicts the running Enrichment Score (ES) graphed versus the rank of the gene when ordered by the *t*-statistic of their differential expression (Untreated vs IFN treatment) result, which is depicted at the bottom.

## References

[bib1] Marçais A, Suarez F, Sibon D, Frenzel L, Hermine O, Bazarbachi A. Therapeutic options for adult T-cell leukemia/lymphoma. Curr Oncol Rep 2013; 15: 457–464. 23943384 10.1007/s11912-013-0332-6

[bib2] Johns TG, Mackay IR, Callister KA, Hertzog PJ, Devenish RJ, Linnane AW. Antiproliferative potencies of interferons on melanoma cell lines and xenografts: higher efficacy of interferon beta. J Natl Cancer Inst 1992; 84: 1185–1190. 1378904 10.1093/jnci/84.15.1185

[bib3] Kinpara S, Hasegawa A, Utsunomiya A, Nishitsuji H, Furukawa H, Masuda T et al. Stromal cell-mediated suppression of human T-cell leukemia virus type 1 expression *in vitro* and *in vivo* by type I interferon. J Virol 2009; 83: 5101–5108. 19264779 10.1128/JVI.02564-08 PMC2682107

[bib4] Oka T, Iwata J, Furihata M, Sonobe H, Miyoshi I, Ohtsuki Y. Inhibitory effects of human interferons on the immortalization of human, but not rabbit, T lymphocytes by human T-lymphotropic virus type-I (HTLV-I). Int J Cancer 1992; 51: 915–920. 1639539 10.1002/ijc.2910510614

[bib5] Moens B, Pannecouque C, López G, Talledo M, Gotuzzo E, Khouri R et al. Simultaneous RNA quantification of human and retroviral genomes reveals intact interferon signaling in HTLV-1-infected CD4+ T cell lines. Virol J 2012; 9: 171. 22917064 10.1186/1743-422X-9-171 PMC3492208

[bib6] Bazarbachi A, Nasr R, El-Sabban ME, Mahé A, Mahieux R, Gessain A et al. Evidence against a direct cytotoxic effect of alpha interferon and zidovudine in HTLV-I associated adult T cell leukemia/lymphoma. Leukemia 2000; 14: 716–721. 10764160 10.1038/sj.leu.2401742

[bib7] Smith D, Buckle GJ, Hafler DA, Frank DA, Höllsberg P. HTLV-I-infected T cells evade the antiproliferative action of IFN-β. Virology 1999; 257: 314–321. 10329542 10.1006/viro.1999.9679

[bib8] Tamura K, Makino S, Araki Y, Imamura T, Seita M. Recombinant interferon beta and gamma in the treatment of adult T-cell leukemia. Cancer 1987; 59: 1059–1062. 2880655 10.1002/1097-0142(19870315)59:6<1059::aid-cncr2820590602>3.0.co;2-m

[bib9] Gill PS, Harrington W, Kaplan MH, Ribeiro RC, Bennett JM, Liebman HA et al. Treatment of adult t-cell leukemia–lymphoma with a combination of interferon Alfa and Zidovudine. N Engl J Med 1995; 332: 1744–1748. 7760890 10.1056/NEJM199506293322603

[bib10] Shimoyama M. Diagnostic criteria and classification of clinical subtypes of adult T-cell leukaemia-lymphoma. a report from the lymphoma study group (1984-87). Br J Haematol 1991; 79: 428–437. 1751370 10.1111/j.1365-2141.1991.tb08051.x

[bib11] Nakahata S, Saito Y, Marutsuka K, Hidaka T, Maeda K, Hatakeyama K et al. Clinical significance of CADM1/TSLC1/IgSF4 expression in adult T-cell leukemia/lymphoma. Leukemia 2012; 26: 1238–1246. 22289924 10.1038/leu.2011.379

[bib12] Manivannan K, Rowan AG, Tanaka Y, Taylor GP, Bangham CRM. CADM1/TSLC1 identifies HTLV-1-infected cells and determines their susceptibility to CTL-mediated lysis. PLOS Pathog 2016; 12: e1005560. 27105228 10.1371/journal.ppat.1005560 PMC4841533

[bib13] Alizadeh Aa, Bohen SP, Lossos C, Martinez-Climent JA, Ramos JC, Cubedo-Gil E et al. Expression profiles of adult T-cell leukemia-lymphoma and associations with clinical responses to zidovudine and interferon alpha. Leuk Lymphoma 2010; 51: 1200–1216. 20370541 10.3109/10428191003728628 PMC4296320

[bib14] Kinpara S, Kijiyama M, Takamori A, Hasegawa A, Sasada A, Masuda T et al. Interferon-α (IFN-α) suppresses HTLV-1 gene expression and cell cycling, while IFN-α combined with zidovudin induces p53 signaling and apoptosis in HTLV-1-infected cells. Retrovirology. Retrovirology;\ 2013; 10: 1. 23688327 10.1186/1742-4690-10-52 PMC3698133

[bib15] Datta A, Bellon M, Sinha-Datta U, Bazarbachi A, Lepelletier Y, Canioni D et al. Persistent inhibition of telomerase reprograms adult T-cell leukemia to p53-dependent senescence. Blood 2006; 108: 1021–1029. 16569765 10.1182/blood-2006-01-0067 PMC1895862

[bib16] Kataoka K, Nagata Y, Kitanaka A, Shiraishi Y, Shimamura T, Yasunaga J et al. Integrated molecular analysis of adult T cell leukemia/lymphoma. Nat Genet 2015; 47: 1304–1315. 26437031 10.1038/ng.3415

[bib17] Sancéau J, Hiscott J, Delattre O, Wietzerbin J. IFN-β induces serine phosphorylation of Stat-1 in Ewing's sarcoma cells and mediates apoptosis via induction of IRF-1 and activation of caspase-7. Oncogene 2000; 19: 3372–3383. 10918594 10.1038/sj.onc.1203670

