# Supplementary material for: IFN-β induces greater antiproliferative and proapoptotic effects and increased p53 signaling compared with IFN-α in PBMCs of Adult T-cell Leukemia/Lymphoma patients
Source: Blood Cancer J. 2017 Jan 27;7(1):e519–. doi: 10.1038/bcj.2016.126 (PMC5301034; doi:10.1038/bcj.2016.126)
Supplement: Supplementary Information [file bcj2016126x1.docx]

**Materials and Methods**

**Ethics**

This study was approved by the Ethics Review Board of “Hospital Universitário Professor Edgar Santos” (registration number 32050106), according to the principles of the Declaration of Helsinki, and all individuals included in this report signed an informed consent form before enrolment. Data handling and processing was additionally approved by the Medical Ethics Commission of the UZ Leuven hospital, Belgium, under registration number s57931.

**Patient recruitment, diagnosis and treatment**

Between 2001 and 2007, a total of 30 leukemia patients were recruited from the “Hospital Universitário Professor Edgar Santos” (HUPES, Federal University of Bahia), inclusion and exclusion criteria have been previously described (1). All cases were confirmed as HIV negative and 26 were diagnosed as clinically definite ATLL according to (2), with serology, inverted PCR and/or flow cytometry carried out as previously described (3). Blood samples were obtained from 22 individuals. Of the examined 22 patients, 7 were classified as acute, 10 as smoldering, 3 as chronic and 2 as lymphoma. The male:female ratio was 1:1.44. The median age was 47,5 years, with a range of 21 to 78 years. Samples were obtained before treatment, survival at five year follow-up data was available for 21 patients, with one lost to follow-up. Patient treatment was in agreement with published international consensus (4) with “watchful waiting” for smoldering forms followed by treatment upon disease progression, IFN+AZT combination therapy for chronic/acute forms and chemotherapy for lymphoma patients. Demographical and clinical details for all patients as well as full experimental assay details can be found in Supplementary Table S1. The clinical details of one patient in this data set have previously been described in a case report (5).

**IFN-α and -β bioactivity determination**

A single batch of clinical grade IFN-α2A (3x10^6^ IU/ml, a gift from Blausiegel Farmacêutica, São Paulo, Brazil) and clinical grade IFN-β1a (1x10^6^ IU/ml, Biogen, Cambridge, Massachusetts, U.S., a gift from Dr. D. Brassat, Toulouse, France) was used throughout the study to eliminate any possible variation in bioactivity during the study period. Stock solutions were prepared in sterile saline and working solutions in RPMI 1640 medium, supplemented with 10% heat inactivated fetal calf serum, 20 μg/ml gentamicin (GIBCO® Invitrogen, Belgium). Bioactivity of IFN-α and IFN-β was determined according to WHO guidelines in order to preclude any potential bias owing to the different antiviral effects of the two interferon types. The antiviral activity of both IFNs was measured against Vesicular Stomatitis Virus (VSV) in Wish cells and showed no statistically significant differences, in agreement with the reports of Sancéau et al. (6).

**Treatment conditions**

Antiviral activity, proliferation and apoptosis were measured as described below in three distinct treatment conditions: either left untreated or stimulated at the start of the experiment with either IFN-α (1000 U/ml) or IFN-β (1000 U/ml), as in (7–9). Neither IL-2 nor PHA was added to the *ex vivo* cultures so that *in vivo* conditions are approximated as closely as possible.

**Proliferation assay**

Peripheral blood mononuclear cells (PBMCs, 1 x 10^6^ cells/ml) were plated in 96-well U bottom plates in RPMI + 10% fetal calf serum (FCS), 200 μl/well in the three conditions and left for 5 days at 37°C and 5% CO2 as in (10). Lymphoproliferation was quantified by [^3^H] thymidine incorporation after a 12-16h pulse with [^3^H] thymidine (1 μCi/well). Incorporation of radioactive label was measured by gas phase scintillation (Direct Beta Counter Matrix 9600, PerkinElmer Life Sciences, MA). Results are expressed as the mean counts per minute in triplicate cultures.

**Apoptosis assays**

PBMCs (1-2 × 10^6^ cells/ml) were plated in 24-well plates in RPMI + 10% FCS, 1 ml/well in the three treatment conditions, for 48h at 37°C and 5% CO2 as in (7,11). Apoptosis resistance of the samples was tested through etoposide and serum starvation (1% FCS) treatment. Active caspase-3 was measured by flow cytometry (FACSort, BD Biosciences, Franklin Lakes, NJ) using a CBA apoptosis kit (BD Biosciences).

**Quantification of HTLV-1 p19 expression**

HTLV p19 protein levels in PBMC 48h culture supernatants were measured in the three treatment conditions, using the HTLV-I/II p19 antigen ELISA (ZeptoMetrix, Buffalo, NY), according to the manufacturer’s instructions.

**Statistical analysis**

Comparison of proliferation, apoptosis and viral protein expression assay results between the different treatment conditions was performed using the nonparametric Friedman rank sum test. Unless otherwise noted, reported p-values for these tests were corrected for multiple testing using the Bonferroni method.

**Microarray analysis**

Total RNA was extracted according to manufacturer’s protocol (QIAgen, Benelux B.V. Venlo, Netherlands) from a total of 20 samples: six patients’ parallel 48h cell cultures in the three treatment conditions plus two additional patients’ untreated control samples. Whole genome microarray was performed at the VIB Nucleomics Facility (Leuven, Belgium) using the GeneChip Human Gene 1.0 ST Array with the WT PLUS reagent kit (Affymetrix, Santa Clara, CA), according to manufacturer’s instructions. Data were RMA preprocessed in R using the Bioconductor oligo package (12). Differential expression analysis was then performed using the Bioconductor limma package (13). A moderated, paired t-test was used to determine differential transcript expression between the three conditions. Genes were accepted as differentially expressed in a condition if their Benjamini-Hochberg corrected p-value was lower than 0.05. Both the preprocessed and raw data from the microarray experiments is available at the National Center for Biotechnology Information Gene Expression Omnibus under accession number GSE85487.

**Gene Set Analysis (GSA)**

GSA was performed in R using the platform for integrative analysis of omics data (piano) package (14). This package implements a range of GSA methods, including Gene Set Enrichment Analysis (GSEA) (15), and provides a consensus score of the GSA results. The C2 canonical pathways and the C5 Gene Ontology Biological Processes gene sets were collected from the Molecular Signatures Database (MSigDB) and tested for enrichment.

**References**

1. Bittencourt AL, Vieira MDG, Brites CR, Farre L, Barbosa HS. Adult T-Cell Leukemia/Lymphoma in Bahia, Brazil. Am J Clin Pathol. 2007 Nov 1;128(5):875–82.

2. Shimoyama M. Diagnostic criteria and classification of clinical subtypes of adult T-cell leukaemia-lymphoma. A report from the Lymphoma Study Group (1984-87). Br J Haematol. 1991 Nov;79(3):428–37.

3. Farre L, Bittencourt AL, Silva-Santos G, Almeida A, Silva AC, Decanine D, et al. Fas 670 promoter polymorphism is associated to susceptibility, clinical presentation, and survival in adult T cell leukemia. J Leukoc Biol. 2007 Sep 17;83(1):220–2.

4. Tsukasaki K, Hermine O, Bazarbachi A, Ratner L, Ramos JC, Harrington W, et al. Definition, Prognostic Factors, Treatment, and Response Criteria of Adult T-Cell Leukemia-Lymphoma: A Proposal From an International Consensus Meeting. J Clin Oncol. 2008 Dec 15;27(3):453–9.

5. Bittencourt AL, Barbosa HS, Requião C, da Silva AC, Vandamme A-M, Van Weyenbergh J, et al. Adult T-cell leukemia/lymphoma with a mixed CD4+ and CD8+ phenotype and indolent course. J Clin Oncol. 2007 Jun 10;25(17):2480–2.

6. Sancéau J, Hiscott J, Delattre O, Wietzerbin J. IFN-β induces serine phosphorylation of Stat-1 in Ewing’s sarcoma cells and mediates apoptosis via induction of IRF-1 and activation of caspase-7. Oncogene. 2000 Jul 17;19(30):3372–83.

7. Moens B, Decanine D, Menezes SM, Khouri R, Silva-Santos G, Lopez G, et al. Ascorbic Acid Has Superior Ex Vivo Antiproliferative, Cell Death-Inducing and Immunomodulatory Effects over IFN-α in HTLV-1-Associated Myelopathy. Carvalho EM, editor. PLoS Negl Trop Dis. 2012 Jul 24;6(7):e1729.

8. Macchi B, D’Onofrio C, Labianca RA, Bonmassar E. Mononuclear cells from peripheral blood of adult donors and from cord blood are equally protected by ??- and ??-interferons against infection with HTLV-I. Pharmacol Res. 1990;22(4):503–14.

9. Feng X, Heyden N Vander, Ratner L. Alpha interferon inhibits human T-cell leukemia virus type 1 assembly by preventing Gag interaction with rafts. J Virol. 2003 Dec;77(24):13389–95.

10. Massoud R, Enose-Akahata Y, Tagaya Y, Azimi N, Basheer A, Jacobson S. Common γ-chain blocking peptide reduces in vitro immune activation markers in HTLV-1-associated myelopathy/tropical spastic paraparesis. Proc Natl Acad Sci. 2015 Sep 1;112(35):11030–5.

11. Van Weyenbergh J, Wietzerbin J, Rouillard D, Barral-Netto M, Liblau R. Treatment of multiple sclerosis patients with interferon-beta primes monocyte-derived macrophages for apoptotic cell death. J Leukoc Biol. 2001;70(5):745–8.

12. Carvalho BS, Irizarry RA. A framework for oligonucleotide microarray preprocessing. Bioinformatics. 2010 Oct 1;26(19):2363–7.

13. Ritchie ME, Phipson B, Wu D, Hu Y, Law CW, Shi W, et al. limma powers differential expression analyses for RNA-sequencing and microarray studies. Nucleic Acids Res. 2015 Apr 20;43(7):e47–e47.

14. Varemo L, Nielsen J, Nookaew I. Enriching the gene set analysis of genome-wide data by incorporating directionality of gene expression and combining statistical hypotheses and methods. Nucleic Acids Res. 2013 Apr 1;41(8):4378–91.

15. Subramanian A, Tamayo P, Mootha VK, Mukherjee S, Ebert BL, Gillette MA, et al. Gene set enrichment analysis: A knowledge-based approach for interpreting genome-wide expression profiles. Proc Natl Acad Sci. 2005 Oct 25;102(43):15545–50.
